# How Flood Damages to Public Infrastructure Affect Municipal Budget Indicators

**DOI:** 10.1007/s41885-017-0015-0

**Published:** 2017-08-19

**Authors:** Christian Unterberger

**Affiliations:** Wegener Center for Climate and Global Change and FWF-DK Climate Change, University of Graz, Brandhofgasse 5, 8010 Graz, Austria

**Keywords:** Flood risk, Flood damage assessment, Extreme events, Adaptation, Disaster risk management, Economic impacts

## Abstract

Countries’ economic activity as well as their fiscal position are vulnerable to climate- and weather related extreme events. Existing research shows that effects on GDP may be either positive or negative, while fiscal implications are clearly negative. Current literature focuses on fiscal implications at the national level. Predicted increases in climate- and weather related extreme events, though, are regionally highly variable. Hence, information concerning the regional vulnerability to specific extreme events is a vital input for adaptation policies. To answer this information demand, this article looks at how flood damages to public infrastructure affect four budget figures (*current income balance, asset management balance, financial transaction balance*, and the *annual result*), exploring the case of Upper Austrian municipalities. Based on a dynamic model and a sample of 442 municipalities from 2009 to 2014 it is found that damages to public infrastructure have a negative impact on municipalities’ *current income* balance and their *annual result*. This indicates a weakening of municipalities’ financial situation. To increase municipalities’ budgetary resilience with regards to public flood damages, municipalities can revert to stricter land use regulation and precautionary measures such as wet- or dry-flood proofing, or to flood insurance.

## Introduction

Patterns of climate- and weather related extreme events have changed and are projected to continue to do so (IPCC 2012). The number of record breaking precipitation events increased globally by 12% between 1981 and 2010, with record-breaking being defined as any daily rainfall (sum) that exceeds all previous observations in the given time series (Lehmann et al. 2015). These observations, however, are subject to high regional variability. In Europe as a whole, for example, the increase amounts to 31%, while at the same time the Mediterranean region experienced a reduction in record-breaking precipitation events by 27%. Regional climate models suggest a continued increase in extreme weather events. Regions may experience an increase in heavy precipitation events even though total precipitation in these regions is projected to decrease (IPCC 2012). By the end of the twenty first century, heavy winter precipitation is projected to increase in central and northern Europe and for north-eastern Europe, a further increase in heavy summer precipitation is expected (Beniston et al. 2007).

The existing literature consistently finds a positive government spending reaction and a worsening of national budgets in response to natural disasters (Leppänen et al. 2015). Estimates provided by Lis and Nickel (2010) show that extreme weather events have a significant negative effect on countries’ budget deficits, ranging from 0.23% to 1.1% of gross domestic products (GDP). Generally, budgetary impacts of extreme weather events have so far only been analyzed at the national level (Ouattara and Strobl 2013; Lis and Nickel 2010; Heipertz and Nickel 2008). Impacts of extreme weather events and climate change, however, do vary at the subnational level and therefore affect regional governments differently (Leppänen et al. 2015).

Effective climate change adaptation and risk management therefore have to be customized to specific regional circumstances (IPCC 2012). In order to implement successful flood risk management, policy makers have to understand the interaction between hazard, exposure and vulnerability (Crichton 2008) and aim to reduce flood risk at the regional level. To achieve this, they need to know where regional exposure, vulnerability, and eventually flood risk are highest i.e. which factors drive existing flood risk and where to reduce risk first. One way to obtain this information is to look at past flood related losses. Generally, there are different kinds of losses one may consider. Most existing studies analyze total economic losses (Hallegatte et al. 2007; Pfurtscheller 2014; Rojas et al. 2013). Other studies look at insured losses (e.g., Czajkowski et al. 2013; Barthel and Neumayer 2012; Changnon 2007, 2009). Typically these studies consider private losses and analyze the impacts these losses have on economic welfare at the national level. What is missing is an analysis of damages to public infrastructure and the effect these damages have on public spending at the regional (i.e. subnational) level.

Damages to public infrastructure may have different repercussions than private losses. They are likely to be more costly (e.g. in EUR/m^2^ terms) as the furnishings and equipment of schools, hospitals, and kindergartens most likely exceed the average value of private furniture (Aerts et al. 2014). They have to be repaired quickly, because public infrastructure and buildings provide services the whole population relies on. They are not accounted for ex ante, and thus, they represent additional liabilities on top of compensation payments to the private sector. Despite these specific features, damages to public infrastructure have so far only been included in overall cost assessments of natural disasters without being differentiated from private damages.

This article applies dynamic panel estimation to analyze the impact flood damages to public infrastructure have on four different budget figures of municipalities located in Upper Austria, Austria. The rationale for choosing this area is that Upper Austria is traversed by six big rivers (Danube, Enns, Inn, Salzach, Steyr and Traun) and is densely populated. Due to its diverse topography it is regularly affected by fluvial as well as pluvial flooding.

The contribution of this article is twofold. First, it explicitly focuses on the budgetary impact of flood damages to public assets in a high income country with an elaborated risk management strategy in place. Second, by analyzing the response of four budget figures to public flood damages at municipal level it provides valuable insights regarding the detailed impacts of these damages at the regional level.

The paper is structured as follows. “The Impact of Extreme Events on GDP, Welfare and Budget Positions” section provides an overview over the existing literature. The “Data” section describes the data and the “Empirical Strategy” section explains the empirical strategy followed. Results are presented in the “Results” section. The “Discussion and Conclusion” section discusses the results and concludes.

## The Impact of Extreme Events on GDP, Welfare and Budget Positions

By now, there is strong scientific consensus that climate change will increase the frequency and severity of extreme weather events (Prein et al. 2017; Coumou et al. 2014). Combined with progressive socio-economic development, significant economic and fiscal consequences are projected, with socio-economic development being the main driving force (Rojas et al. 2013; Hallegatte et al. 2011). The consequences range from direct damages extreme events cause, to eventual effects on countries’ GDP and welfare (indirect damages). As for the direct damages, the majority of studies found that losses caused by extreme weather events have increased and are projected to continue to do so in the future, with some regions being more affected than others (Alfieri et al. 2016; Barthel and Neumayer 2012; Hallegatte 2015).

Effects on countries’ GDP and welfare arise as the losses caused by the extreme events impact the supply as well as the demand side of affected economies. These economy-wide effects typically are referred to as indirect damages (Hallegatte 2015). To identify them, existing studies follow two main approaches - econometric approaches and model based approaches. Econometric approaches focus on a series of past events and analyze the mean effect of extreme events by referring for example to the average impact on economic growth. Analyses are conducted on national as well as on regional levels. For the national level, Hochrainer-Stigler (2009), Noy (2009), Jaramillo (2009) and Raddatz (2009) conclude that natural disasters have an adverse impact on economic growth. Earlier studies by Albala-Bertrand (1993) and Skidmore and Toya (2002) report a positive reaction of economic growth in response to natural disasters. According to Cavallo and Noy (2010) and Loayza et al. (2012) this contradiction is attributable to size differences of the analyzed events. While small-scale disasters potentially trigger economic growth, large-scale events tend to have negative impacts. This claim is further confirmed by Noy and Vu (2010), who analyze the impacts of disasters at the province level in Vietnam. It is discovered that more lethal disasters result in lower output growth, whereas disasters that primarily destroy capital and property lead to a short run increase in economic activity.

Model based approaches to assess the economic costs of natural disasters rely on input-output models or computational general equilibrium (CGE) models. Hallegatte (2008) for instance, applies a regional input-output model to assess the indirect losses hurricane Katrina caused in Louisiana in 2005. He discovers that regional indirect losses and regional direct losses have a non-linear relationship with indirect losses increasing disproportionally when a certain threshold in direct damages is passed. Analyzing the macroeconomic impacts of climate change for Austria, Steininger et al. (2016) apply a CGE model that distinguishes 41 sectors. For each of the sectors, specific climate change impacts are implemented, which eventually have direct and indirect effects; the latter being the result of sectoral linkages. It is found that by 2050 climate change has a negative net effect of −0.15% of Austrian GDP. In addition to GDP effects, Steininger et al. (2016) also consider welfare effects and conclude that net welfare losses are three to four times higher than GDP effects.

While the analysis and estimation of losses and economic consequences of natural disasters already are subject of many studies, the fiscal effects of natural disasters only more recently started to attract attention. Ouattara and Strobl (2013) look at the spending reaction in response to hurricanes in Caribbean countries and discover a positive reaction that persists for up to two years. Based on case studies for the EU and the US, Heipertz and Nickel (2008) conclude that the budgetary impact of extreme weather events amounts to an increase in budget deficits by 0.3–1.1% of countries’ GDP. This finding is supported by Lis and Nickel (2010) who find that extreme weather events increase budget deficits by 0.23–1.1% of countries’ GDP, subject to the development level of the country. These results indicate that there is considerable country specific variation when it comes to the disaster elasticity of public spending, which primarily is attributable to the severity of the disastrous events as well as the size of the economy (Heipertz and Nickel 2008).

Leppänen et al. (2015) perform a regional (i.e., subnational) analysis looking at the impact of changes in average regional temperature and precipitation on regional expenditure in Russia. They conclude that temperature increases lead to a reduction in public expenditures, although with decreasing magnitude for warmer regions.

## Data

### Municipalities and Damage Data

**Fig. 1:**
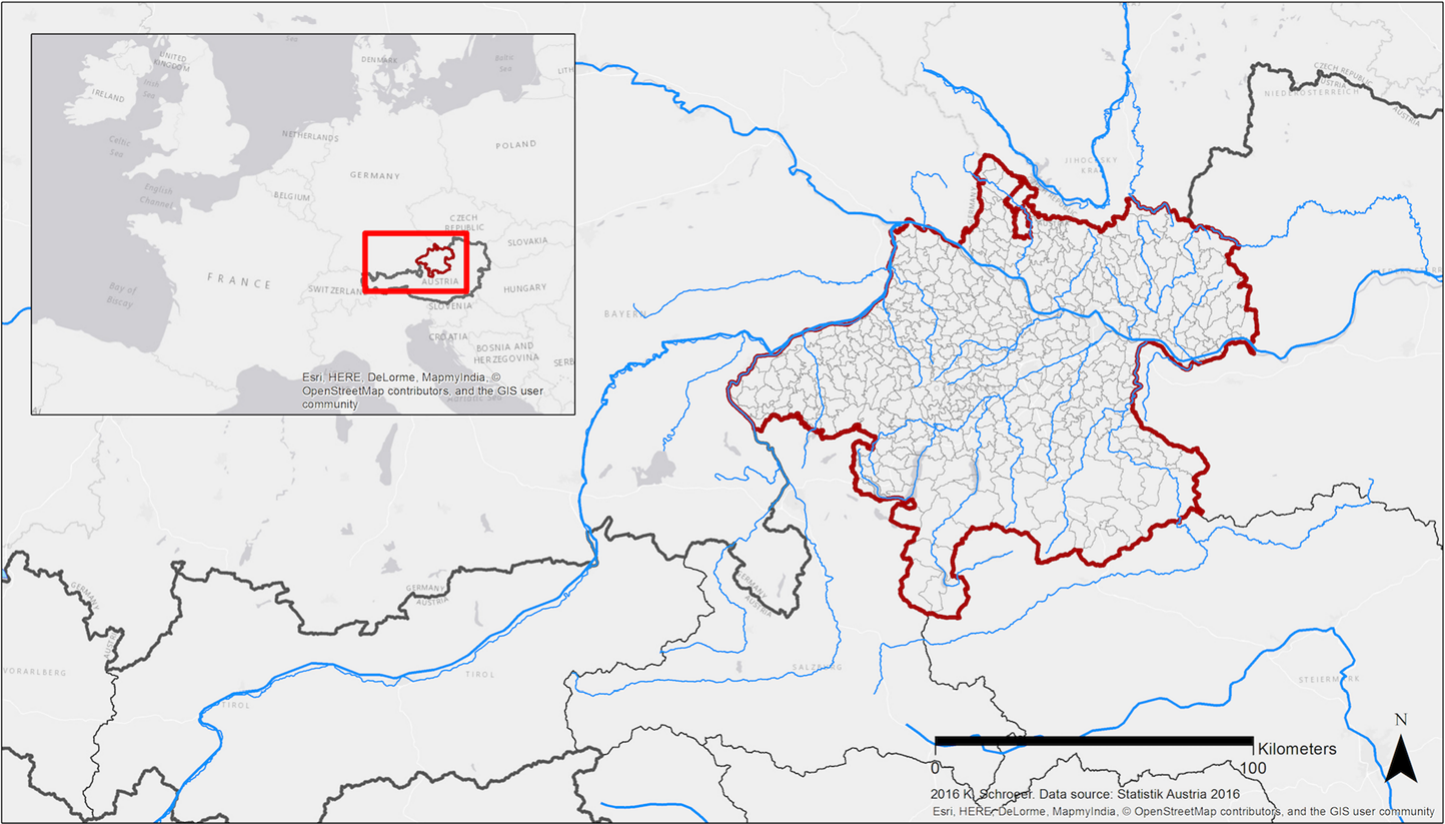
Upper Austria is one of nine Austrian federal states and located in the north of the country (red line). It contains 442 municipalities and is traversed by many rivers, among which the six biggest are the rivers Danube, Enns, Inn, Salzach, Steyr and Traun

The study area focused on is Upper Austria, which is located in the north of Austria (Fig. 1). Upper Austria is one of nine Austrian federal states. It is densely populated and traversed by six major rivers (Danube, Enns, Inn, Salzach, Steyr and Traun). Overall it counts 442 municipalities of which 85% have less than 5000 inhabitants. Upper Austria is regionally diversified with economic hubs like the cities of Linz and Wels on the one hand and rural areas with predominantly agricultural production and tourism as for example the Mühlviertel on the other hand. Consequently the regional GDP per capita has high variability; ranging from EUR 48,900 per capita (Linz-Wels) to EUR 24,900 (Mühlviertel) in 2014 (Statistik Austria 2017).

The variables of interest are flood damages to public infrastructure as reported to the Austrian Disaster Fund by the municipalities of Upper Austria from 2009 to 2014 and budget data. Flood damage data was collected and provided by the Austrian Institute of Economic Research and is available annually. In addition to flood damages also damages to public infrastructure caused by other events (e.g., rock fall, hail, etc.) are included in the analysis. The minimum flood damage reported amounts to EUR 5000 and the maximum reported flood damage is EUR 504,036. The minimum reported damage caused by events other than floods amounts to EUR 1748 and the maximum here is EUR 222,032. No official minimum damage threshold for reporting exists and all reported damages are included in the analysis. Due to administrative burdens it is possible that small damages are less frequently reported. The reported damages are assumed to be correct, since they are verified by authorized experts.

**Fig. 2:**
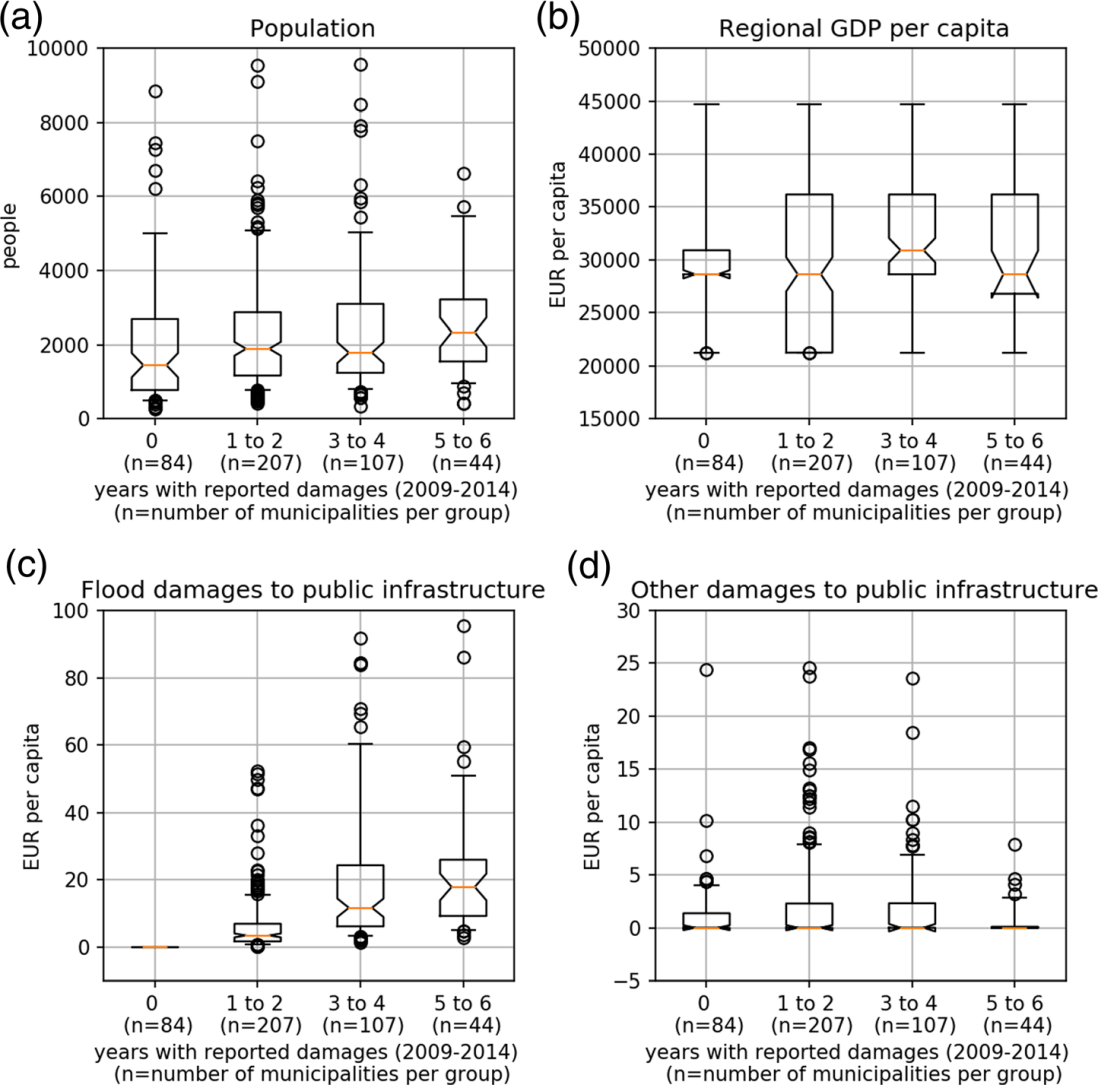
Municipal characteristics grouped by the frequency of reported flood damages to public infrastructure. The names of the four groups on the x-axis indicate the number of years in which damages were reported. The orange line shows the median, the whiskers range from the 9th to the 91st percentile. Outliers are represented by black circles. Units are shown next to the y-axis

Considering different population figures, varying economic activity and geographic location, exposure as well as vulnerability may vary across municipalities. This is shown in Fig. 2a–d. Municipalities are partitioned into four groups, according to the number of years they reported flood damages to public infrastructure between 2009 and 2014. Within this period 84 municipalities never reported any damage *(0, group 1).* Two hundred seven municipalities reported damages in one or two years *(1–2, group 2)* and 107 municipalities reported damages in 3 or 4 years *(3–4, group 3)*. Forty-four municipalities were affected nearly every year, reporting damages five or six times *(5–6, group 4)*. For each group, *population*, *regional GDP* per capita, *flood-* and *other damages to public infrastructure* are shown.

Visual inspection of panel (a) in Fig. 2 suggests that *population numbers* are similar across groups. The same holds true for *regional GDP* per capita (panel (b) of Fig. 2). Hence, neither of these characteristics point to any difference across the four groups. Interestingly, panel (c) reveals that the highest *flood damages* are reported by group 3 and group 4, those municipalities that are affected most frequently. Finally panel (d) shows that municipalities from group 4 report the fewest *other damage* events. This indicates that flood risk and natural disaster risk in general varies regionally.

Other studies use climate change signals or different disaster metrics (e.g. return period for floods or wind speed and duration for hurricanes) to explain damages or budgetary impacts (Czajkowski et al. 2013; Leppänen et al. 2015; Ouattara and Strobl 2013). This study, however, directly links reported flood damages to budgetary indicators. This strategy is taken due to the following reason. Hazards or changes in their occurrence or magnitude only pose a risk when they meet vulnerable exposure (Crichton 2008). With respect to budgetary impacts only the magnitude of the materialized risk (i.e., the reported damages) is important, whereas the magnitude of the hazard event (e.g., return period) is of secondary interest.

### Budget Data

Municipalities’ budgets contain around 70 earning and spending specific sub- positions, which are aggregated to 3 balances *(current income balance*, *asset management balance* and *financial transactions balance*), which together represent the *annual result* of a municipality before transfers between ordinary and extraordinary budget. In order to capture the budgetary impacts of flood damages to public infrastructure, the responses of the three balances as well as the responses of the *annual result* to flood damages are analyzed.

The *balance of current income* is the difference between municipalities’ current revenues and current expenses. Current revenues are generated by means of tax and fee collection, revenues from property and economic activity as well as transfer payments from public bodies (i.e. the federal states). Current expenses are comprised by the payment of wages, administrative- and maintenance expenses and transfer payments to public bodies (i.e. apportionment for hospitals and social security, transfers to the federal state) (Klug 2011). Hence, the *balance of current income* can be considered as municipalities’ cash flow (Schauer 2016). As for the impact of flood damages to public infrastructure it is expected that they have a negative impact on the *current income balance*. First, flood damages potentially restrict municipalities’ ability to collect revenues from property and economic activity. At the same time flood damages are expected to increase current expenses (e.g. maintenance expenses).

The *asset management balance* consists of revenues and expenses generated by asset management activities. On the revenue side, there are sales of assets and rights as well as capital transfers from public bodies. Acquisitions of assets and rights as well as capital transfers to public bodies are to be found on the expenditure side (Schauer 2016). In the short term, capital transfers from public bodies (i.e. investment grants) might increase in order to support stressed municipalities. In the medium to long term municipalities could purchase properties and create retention areas. Overall, small positive effects could be expected.

*The financial transaction balance* is the difference between revenues and expenditures generated by financial transaction activities. The sale of securities, borrowing and the withdrawal of reserves constitute the revenue side. On the expenditure side, there are acquisitions of shares, repayments of loans and the accumulation of reserves (Schauer 2016). Municipalities that suddenly face flood damages could postpone repayments in order to create additional financial leeway. This would reduce the expenditure side of the *financial transaction balance*. At the same time it is very likely that municipalities cover the additional costs by means of borrowing or via the withdrawal of reserves. The overall effect of flood related damages to public infrastructure on the *financial transaction balance* therefore is ambiguous.

The *annual result* before transfers between ordinary and extraordinary budget shows if municipalities generate an overall surplus or an annual deficit. Assuming that flood damages to public infrastructure represent unplanned and additional expenses it is expected that they negatively affect the *annual result*. The *annual result* is a central determinant of the Maastricht result municipalities have to report according to the Austrian Stability Pact (Schauer 2016).

**Fig. 3:**
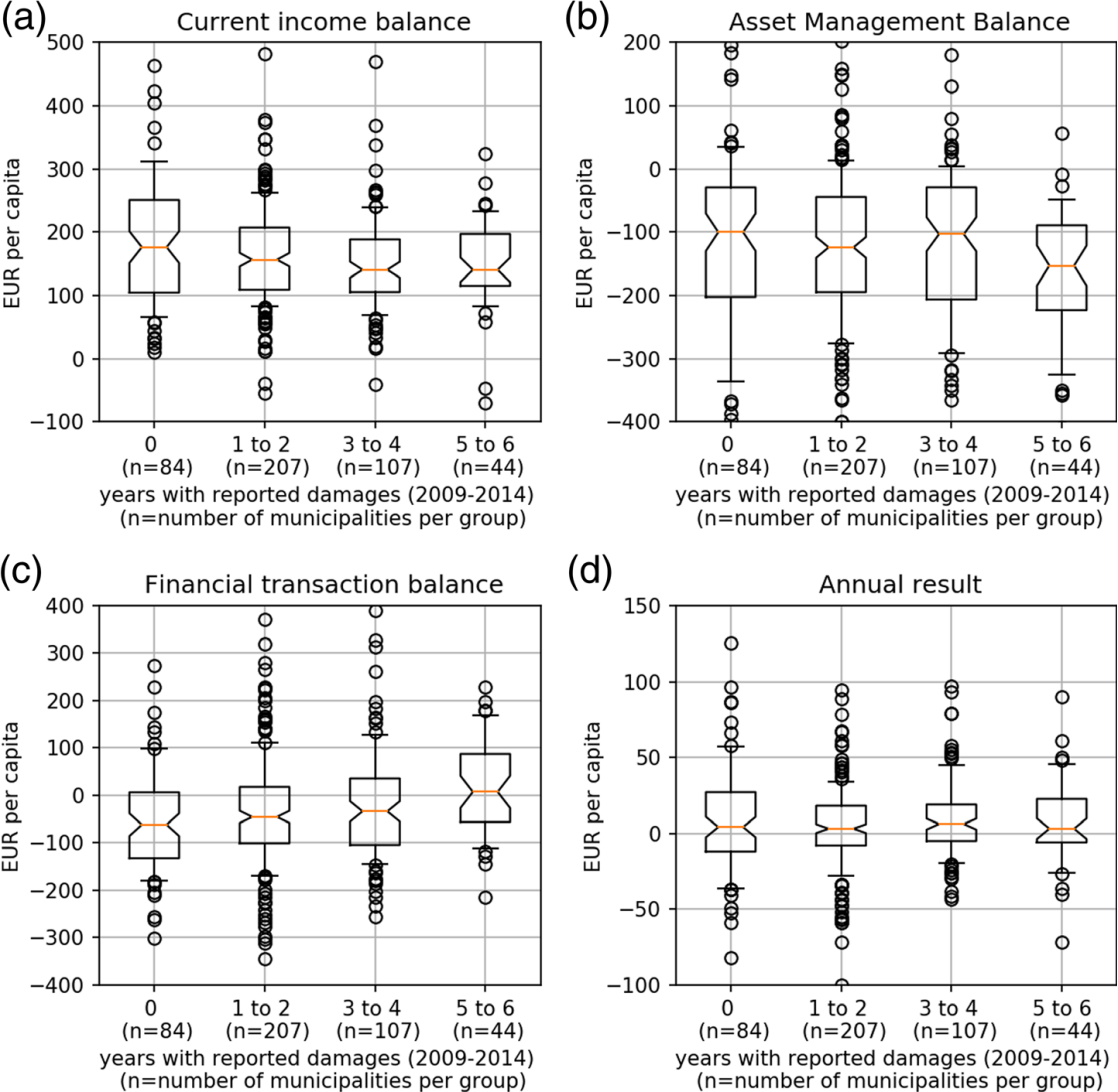
The four budget figures analyzed grouped by the frequency of reported flood damages to public infrastructure. The names of the four groups on the x-axis indicate the number of years in which damages were reported. The orange line shows the median, the whiskers range from the 9th to the 91st percentile. Outliers are represented by black circles. Units are shown next to the y-axis

Figure 3 provides a visualization of the arguments made. As in Fig. 2, municipalities are partitioned into four groups according to the number of years they reported damages between 2009 and 2014. For each group the *balance of current income,* the *asset management balance,* the *financial transaction balance* and the *annual result* are shown.

Panel (a) highlights that municipalities of group 3 and group 4 have a lower *current income balance,* implying that they have a lower cash flow than municipalities that are less frequently affected. Also the *asset management balance* shows variation across groups (panel (b) Fig. 3). As for the *financial transaction balance,* municipalities of group 4 display higher balances (panel (c)). This implies either a reduction in the repayment of loans, an increase in the depletion of reserves or an increase in credits raised. Eventually panel (d) in Fig. 2 shows no discernible difference among the groups regarding the *annual result.*

## Empirical Strategy

To estimate the budgetary impact of extreme weather events, above described data is arranged in a panel data format covering 442 municipalities for a sample period from 2009 to 2014. The estimated model is as follows:

1 $$ {b}_{it}=\alpha +\beta {b}_{i\ t-1}+{\gamma}_1\ F{D}_{it}+{\gamma}_2\ F{D}_{i,t-1}+{\delta}_1O{D}_{it}+{\delta}_2O{D}_{i,t-1}+{\varepsilon}_1{GDP}_{i,t}+{\varepsilon}_2{GDP}_{i,t-1}+\rho t+{\mu}_i+{u}_{it} $$

Equation (1) is estimated separately for each budget indicator, with the dependent variable *b*_*i* , *t*_ of municipality *i* at time *t* referring to the three budget balances mentioned above (*current income balance*, *asset management balance* and *financial transactions balance*), as well as the *annual result* for municipality *i* at year *t*. *b*_*i* , *t* − 1_ refers to the first lag of the endogenous variable. Flood damages to public infrastructure reported by municipality *i* in year *t* are captured by *FD*_*it*_. To account for potential lagged effects reported flood damages have on budget indicators, the first lag of reported flood damages *FD*_*i* , *t* − 1_ is included. Damages to public infrastructure in municipality *i* in year *t* caused by other disastrous events (e.g., rock fall, hail, etc.) are captured by *OD*_*it*_, with *OD*_*it* − 1_ accounting for potential lagged effects. *GDP*_*i* , *t*_ describes regional per capita GDP of municipality *i* at year *t*, and *GDP*_*i* , *t* − 1_ describes the respective first lag. Time is accounted for by the variable *t*. Municipality specific effects such as the geographic position and therewith connected time-invariant factors are denoted as *μ*_*i*_. The error term is represented by *u*_*i* , *t*_.

Generally, the inclusion of control variables is a rather challenging task, given that yearly (economic) data is hardly available at municipal level. To account for different economic development over the years, regional annual per capita GDP is included in the analysis. Regional GDP at NUTS 3 level is available from Statistics Austria (Statistik Austria 2017). Each municipality is located in one out of five NUTS 3 regions (Innviertel, Linz-Wels, Mühlviertel, Steyer-Kirchdorf and Traunviertel). Additionally, the time variable indicates the year of the observation. Clearly, it would be desirable to have more control variables, as it is likely that additional factors influence the analyzed budget indicators. It is believed, however, that including the lagged value of the dependent variable covers the majority of the unavailable information. The estimation method applied (and further described in “Dynamic Panel Methods ”section) is specifically designed for dynamic processes with a small number of available time periods (the panel is “small T and large N”) and potentially endogenous regressors (Roodman 2006).

All variables are log transformed and expressed in EUR/capita terms. This potentially causes problems, as the three budget balances do not necessarily have to be positive (as obvious from Fig. 3). Therefore, the budget variables also were Box-Cox transformed in order to control the accuracy of the log transformation. Both results are similar in size and direction, therefore only the results for the log- transformed variables are reported.

In estimating the budgetary impacts of flood damages to public infrastructure for the 442 municipalities between 2009 and 2014 the typical issues encountered in panel data analysis arise. First, pooled OLS estimation would lead to biased estimators, as it first assumes strict exogeneity (*E*[*u*_*it*_| *X*_*i*1_, …, *X*_*iT*_, *μ*_*i*_] = 0 ,  *with i* = 1 , … , *N and t* = 1 , … , *T*) and second fails to take advantage of the panel structure of the data and ignores time and entity fixed effects. While fixed and random effects regression allow the consideration of entity and time fixed effects, the assumption of strict exogeneity still applies to these estimation methods (Cameron and Trivedi 2005). The possibility of studying dynamics, however, is one of the main advantages when applying panel data. Particularly with respect to budget data it is very likely that a certain degree of persistence exists (i.e., lagged endogenous variables contain valuable information) and that there are feedbacks to exogenous variables (e.g., flood damages).

One potential feedback for example exists between the reported flood- (or other-) damages and spending groups that contain investments for protection measures. If for example municipalities take flood protection measures, invest in slope stabilization, or purchase properties in order to create retention areas, these costs will affect certain budget groups. At the same time, however, they also (hopefully) reduce or even avoid the damages eventually experienced. Ideally, data for expenditures on protection measures would be available. Unfortunately, for the time being this is not the case.

An alternative to the strict exogeneity assumption is weak exogeneity (*E*[*u*_*it*_| *X*_*it*_, *μ*_*i*_] = 0 ,  *with i* = 1 , … , *N and t* = 1 , … , *T*), hence to allow for lagged endogenous variables and feedbacks and thereby establish a dynamic model. In a dynamic panel model lagged endogenous regressors are included. This inclusion, however, violates a random effects assumption, as the lagged endogenous variable is not independent from the time fixed effects *μ*_*i*_. On these grounds it is more convenient to estimate dynamic panels with fixed effects methods (Cameron and Trivedi 2005). In what follows the dynamic panel method is briefly explained.

### Dynamic Panel Methods

To simplify matters, dynamic panel models here are described without the inclusion of regressors. Consider the following dynamic panel model in which this year’s budgetary variable *b*_*it*_ only depends on its value in the previous year *b*_*i* , *t* − 1_, a municipality fixed effect *μ*_*i*_, and an error term *u*_*i* , *t*_:

2 $$ {b}_{it}=\gamma {b}_{i,t-1}+{\mu}_i+{u}_{i,t}\  with\ i=1,\dots, N\  and\ t=1,\dots, T $$

When estimating dynamic panel models, first differences are taken to eliminate the fixed effects. In doing so the municipality fixed effect disappears and the first difference of the endogenous variable is regressed on its second difference and the first difference of the error term.

3 $$ {b}_{i,t}-{b}_{i,t-1}=\gamma \left({b}_{i,t-1}-{b}_{i,t-2}\right)+{u}_{i,t}-{u}_{i,t-1} $$

This specification, however, cannot be estimated by OLS as *E*[*u*_*i* , *t*_ − *u*_*i* , *t* − 1_| *b*_*i* , *t* − 1_ − *b*_*i* , *t* − 2_] ≠ 0. The first difference estimator is biased and inconsistent. However, if it is assumed that the error term *u*_*i* , *t*_ is not serially correlated, *b*_*i* , *t* − 2_ is correlated with( *b*_*i* , *t* − 1_ − *b*_*i* , *t* − 2_), and uncorrelated with the error term (*u*_*i* , *t*_ − *u*_*i* , *t* − 1_). The variable *b*_*i* , *t* − 2_ can be used as instrumental variable for (*b*_*i* , *t* − 1_ − *b*_*i* , *t* − 2_). This leads to an equation system, consisting of Eqs. 3 and 4, which can be solved with two-stage least square estimation to obtain a consistent estimator for *γ* (Anderson and Hsiao 1982).

4 $$ {b}_{i,t-1}-{b}_{i,t-2}=\alpha {b}_{i,t-2}+{v}_{i,t} $$

(Arellano and Bond 1991) propose a more general framework to estimate dynamic panel models. Applying general methods of moment estimation (GMM) they show that plenty instrumental variables exist for moment conditions in the GMM framework. Provided that there is no autocorrelation in the error term, $\left(T-1\right)\frac{T}{2}$ moment conditions exist to estimate *γ*. The Arellano Bond estimator is implemented in STATA via the xtabond2 routine (Roodman 2006). This estimation process controls for the problems discussed above (e.g. incorporation of lagged endogenous variables, endogeneity) and is therefore well suited to identify the impacts flood related damages to public infrastructure have on municipalities’ budget figures (Roodman 2006). The results of the analysis are shown in the following “Results” section.

## Results

As explained in the “Data” section, the impact of flood damages on four different budget positions *(current income balance, asset management balance, financial transaction balance,* and *annual result*) of Upper Austrian municipalities is estimated by means of dynamic panel estimation. The results of these estimations as well as the number of observations and test statistics are summarized in Table 1. To account for heteroskedasticity and autocorrelation, robust estimation is applied. In this case the Sargan test is inconsistent and the Hansen statistic tests for the exogeneity of instruments (Roodman 2006). The tests for autocorrelation show that generally there is first-order serial correlation but no second order serial correlation. This indicates that the choice of instruments used is appropriate. Furthermore, the Wald test rejects the hypothesis that the included parameters are simultaneously equal to zero.

**Table 1:** Results, number of observations, and test statistics of the dynamic panel estimation for the impacts damages to public infrastructure have on budget indicators

	Current income balance (in €/capita)	Asset management balance (in €/capita)	Financial transaction balance (in €/capita)	Annual result (in €/capita)
L1. Dependent Variable	0.168*** (0.031)	0.103** (0.045)	0.158*** (0.033)	−0.127*** (0.026)
Flood damages to public infrastructure	−0.037** (0.018)	0.011 (0.059)	−0.013 (0.075)	−0.218*** (0.068)
L1. Flood damages to public infrastructure	−0.024 (0.016)	0.037 (0.054)	−0.014 (0.666)	−0.164*** (0.063)
Other damages to public infrastructure	−0.023 (0.044)	0.083 (0.115)	−0.102 (0.129)	−0.284*** (0.109)
L1. other damages to public infrastructure	−0.009 (0.037)	−0.028 (0.072)	−0.021 (0.083)	−0.114 (0.089)
Regional GDP	7.263*** (0.724)	5.279*** (1.883)	−11.509*** (2.679)	10.946*** (2.429)
L1. Regional GDP	−6.936*** (0.734)	9.001*** (3.322)	−6.777*** (4.590)	-21.238*** (3.999)
Time	0.162*** (0.017)	−0.819*** (0.267)	0.868** (0.348)	0.706** (0.345)
Number of Observations	1768	1768	1768	1768
Wald chi2 (10) p > chi2	0.000	0.000	0.000	0.00
Autocorrelation test AR(1) p > chi2	0.000	0.000	0.000	0.00
Autocorrelation test AR(2) p > chi2	0.353	0.379	0.607	0.572
Hansen test p > chi2	0.000	0.992	0.111	0.628

The coefficients show the percentage change of the dependent variable for a 1% change in the independent variable. The standard deviation is given in brackets. The asterisks show the significance level where * indicates *p* < 0.1, ** indicates *p* < 0.05 and *** indicates *p* < 0.01

When interpreting the coefficients in Table 1, it has to be kept in mind that the dependent as well as the independent variables are log transformed. Therefore the coefficients on the lagged dependent variable, the flood and other damages to public infrastructure as well as the regional GDP indicate the elasticity of the respective budget position with respect to the dependent variable.

Looking at the estimated impacts of flood damages to public infrastructure, *current income balances* reveal a significant negative response, suggesting that flood damages directly and negatively affect municipalities’ *current income balances*. The negative coefficient of −0.037 indicates that a 1% increase in the average flood related public infrastructure damages leads to a decrease of the *current income balance* by around 0.04%. The coefficient on the lagged flood damages is not significant, suggesting that the overall effect on the *current income balance* subsides within the year the damage event is reported. It is important to recognize that the average public flood damage is exceeded manifold in case floods happen, the reason being that the dataset also contains municipalities, which are never or only slightly affected by floods. The maximum flood damages reported amounts to EUR 1380 per capita, whereas the mean is around EUR 10 per capita. Looking at the upper 95th percentile of reported damages i.e. damages that amount to EUR 50 per capita would represent a 400% increase as compared to the mean. Looking at the test statistics provided in Table 1, the Hansen statistic displayed in the first column suggests that the set of instruments used for the first regression are not appropriate. Nevertheless, the results are presented and explained for the following reasons. First, the tests for autocorrelation show the expected results, indicating that the residuals are not serially correlated of order 1, hence fulfill the conditions. Second, (Roodman 2006) states that the Hansen test should not be relied upon too faithfully as it gets weaker the more moment conditions there are.

Apparently flood damages to public infrastructure have no significant effect on the *asset management balance*. This observation may be explained by the fact that determinants of the expenditure and revenues side of the balance experience similar responses (i.e. both decrease or increase), which eventually neutralize each other when it comes to the overall effects. The same argument is applicable to the results obtained for the *financial transaction balance*. None of the coefficients on flood damages to public infrastructure exhibits significance, indicating that the overall balance remains unaffected by flood damages. Both results are supported by the Autocorrelation- and the Hansen test.

Unlike the *asset management*- and *financial transaction balance*, the *annual result* is significantly affected by flood damages to public infrastructure. Here, a 1% increase in reported damages leads to a deterioration by 0.22% within the same year. This impact is found to be persistent, as indicated by the significant negative coefficient on the first lag of the flood damages. Interestingly, also damages to public infrastructure caused by other events (e.g. rock fall, hail, etc.) are found to directly negatively affect municipalities’ *annual result*. A 1% increase in damages leads to a reduction by 0.28%. Unlike flood damages, however, other damages do not have a lasting effect on municipalities’ *annual result*, as shown by the insignificant coefficient on the first lag.

Looking at the other explanatory variables, the coefficient on the lag of each dependent variable shown in line 1 of Table 1 reveals that the budget positions significantly depend on their past values. *Current income-, asset management*- and *financial transaction balance* all show significant positive impacts of the lagged values. The negative coefficient on the *lagged annual result* suggests that municipalities try to balance their *annual result* over the years. As for the coefficients on regional GDP statistically significant positive effects are discovered for the *current income balance*, the *asset management balance*, and municipalities’ *annual result*. Both, the *current income balance* as well as the *annual result* exhibit a positive response to changes in current regional GDP per capita and a negative response to previous year’s. The *financial transaction balance* shows significant negative responses for the current as well as the lagged regional GDP per capita.

## Discussion and Conclusion

The analysis shows that flood damages to public infrastructure negatively affect municipalities’ *current income balance* and *annual result*. While the negative effect on *the current income balance* subsides within the year the damage event is reported, the impact on the *annual result* is more persistent. It is still observed one year after the reported damage. Neither the *asset management balances* nor the *financial transaction balances* show any response to flood damages to public infrastructure.

The observation that the *current income balance* is only directly affected can be explained by looking at its composition. As mentioned in “Budget Data” section current expenses consist among other things of administrative and operating expenses. It is conceivable that these expenses increase in direct response to flood damages to public infrastructures. At the same time also the current earnings potentially increase, for example due to an increase in transfer payments in response to flood damages. The significant negative coefficient indicates that for *the current income balance* the increase in current expenditures prevails. To explain the insignificant effects flood damages have on the *asset management balance*, again a look at its composition is helpful. On its earning side, flood damages to public infrastructure could for example reduce the revenues generated by the disposition of property and rights. On its expenditure side, flood damages potentially negatively affect capital transfer payments from the municipality to other public bodies. Overall, these effects might cancel each other out, eventually leaving the *asset management balance* without any significant changes in response to flood damages. The same line of reasoning applies to the results obtained for the *financial transaction balance*.

Given the fact that flood damages to public infrastructure have a persistent impact on *municipalities’ annual result* it is interesting to know for how long the negative effect is observable. Therefore, the estimation model presented in Eq. 1 was extended to include two lags of flood- and other damages to public infrastructure. The amended estimation model, as well as the results, are shown in the Appendix. Again, the *current income balance* is found to be significantly negatively affected by flood damages to public infrastructure. As before, this effect subsides within the year the damage was reported. Looking at municipalities’ *annual result*, the results obtained reveal that the second lag has no significant effect. Hence, the negative impact of flood damages to public infrastructure does not manifest beyond one year after the event.

Generally the results indicate that flood damages to public infrastructure have an adverse effect on municipalities’ budgets. The deterioration of the *current income balance* implies that less funds are available to finance investments, repay loans or build up reserves (Schauer 2016). Additionally, the negative response of municipalities’ *annual result* indicates that the adverse effect does not only directly affect operating business but also has a prolonged effect on municipalities’ overall financial situation.

The results obtained for the budgetary response to extreme events on municipal level are in line with those presented by existing studies at country level. Ouattara and Strobl (2013) report a positive spending effect in response to hurricanes in Caribbean countries and Lis and Nickel (2010) discover a worsening of the budget balance (i.e. an increase in spending) in response to natural disasters.

In order to contain the damage potential floods bring along, municipalities have to actively reduce their exposure. This can be achieved via four different ways: (I) an increase of flood protection levels, (II) a reduction of peak flows, (III) reduction of vulnerabilities, and (IV) relocation (Alfieri et al. 2016). As shown by Alfieri et al. (2016) the implementation of these measures (particularly peak flow reduction and higher flood protection) has the potential to reduce flood risk significantly. At regional level particularly adaptation by means of non-structural measures is found to be important. This includes for example stricter land use regulation or the enhancement of precautionary measures such as dry- or wet proofing, the installment of backflow preventers or the adaptation of building use and interior equipment (Thieken et al. 2016; de Moel et al. 2014). Clearly adapting to flood risk also comes with costs, however, as shown by Rojas et al. (2013), there are many measures for which the avoided damages do warrant the efforts. Particularly so because more resilient budgets at municipality level also increase the resilience of superior budgets i.e. the budgets of the federal and the national state.

Additionally, municipalities could consider insuring themselves against potential flood damages. As proposed by Kousky and Shabman (2015), municipalities might engage in parametric community insurance schemes with capped coverage levels. While this insurance scheme would naturally also cover private property damages it would still incentivize municipalities to engage in prudent spatial planning and guarantee that building codes are satisfied, otherwise municipalities would not be granted access to the scheme. This approach would incentivize risk reduction in the public as well as the private domain.

For future research the results of this study promote the following directions. First, the exposure of public infrastructure is an important determinant of the overall disaster risk faced by the public sector and therefore needs to receive more attention. Second, (regional) data on adaptation spending is needed so as to analyze to what extent adaptation measures isolate (regional) budgets from the impacts of natural disasters and extreme events. Third, the projected increase in extreme precipitation events (Prein et al. 2017) also carries implications with regards to suitable adaptation measures. Hence a differentiation between the different processes that cause flood damages (fluvial vs. pluvial) is vital so as to provide more precise risk assessments.

## Appendix

Equation 5 estimates the effects flood damages to public infrastructure have on budget indicators by including 2 lags of the damage variables. Results are provided in Table 2

5 $$ {b}_{it}=\alpha +\beta {b}_{i\ t-1}+{\gamma}_1\ F{D}_{it}+{\gamma}_2\ F{D}_{i,t-1}+{\gamma}_3\ F{D}_{i,t-2}+{\delta}_1O{D}_{it}+{\delta}_2O{D}_{i,t-1}+{\delta}_3O{D}_{i,t-2}+{\varepsilon}_1{GDP}_{i,t}+{\varepsilon}_2{GDP}_{i,t-1}+\rho t+{\mu}_i+{u}_{it} $$

**Table 2:** Results, number of observations, and test statistics of the dynamic panel estimation for the impacts damages to public infrastructure have on budget indicators

	Current income balance (in €/capita)	Asset management balance (in €/capita)	Financial transaction balance (in €/capita)	Annual result (in €/capita)
L1. Dependent Variable	0.144*** (0.034)	0.111** (0.018)	0.132*** (0.000)	−0.157*** (0.000)
Flood damages to public infrastructure	−0.048** (0.020)	0.004 (0.961)	−0.060 (0.527)	−0.181** (0.090)
L1. Flood damages to public infrastructure	−0.016 (0.018)	0.009 (0.916)	−0.043 (0.602)	−0.118* (0.085)
L2. Flood damages to public infrastructure	−0.018 (0.019)	−0.070 (0.247)	−0.044 (0.549)	−0.002 (0.075)
Other damages to public infrastructure	−0.034 (0.039)	0.116 (0.475)	0.052 (0.774)	−0.239* (0.139)
L1. other damages to public infrastructure	0.019 (0.032)	−0.074 (0.489)	0.031 (0.750)	−0.205 (0.114)
L2. other damages to public infrastructure	0.001 (0.029)	−0.052 (0.507)	0.024 (0.797)	0.004 (0.092)
Regional GDP	5.209*** (0.912)	4.470 (0.395)	7.303 (0.130)	8.394* (4.765)
L1. Regional GDP	−4.830*** (0.930)	6.169 (0.276)	−15.156*** (0.003)	−20.962*** (5.419)
Time	0.094*** (0.021)	−0.600** (0.017)	0.6347** (0.017)	0.732** (0.321)
Number of Observations	1326	1326	1326	1326
Wald chi2 (10) p > chi2	0.000	0.000	0.000	0.00
Autocorrelation test AR(1) p > chi2	0.000	0.000	0.000	0.00
Autocorrelation test AR(2) p > chi2	0.852	0.382	0.215	0.320
Hansen test p > chi2	0.005	0.983	0.183	0.601

The coefficients show the percentage change of the dependent variable for a 1% change in the independent variable. The standard deviation is given in brackets. The asterisks show the significance level where * indicates *p* < 0.1, ** indicates *p* < 0.05 and *** indicates *p* < 0.01
